# *Flammulina velutipes* mycorrhizae dietary fiber attenuates the development of obesity via regulating lipid metabolism in high-fat diet-induced obese mice

**DOI:** 10.3389/fnut.2025.1551987

**Published:** 2025-03-12

**Authors:** Fengjuan Jia, Yulan Gao, Jian Zhang, Furong Hou, Junyan Shi, Shasha Song, Shifa Yang

**Affiliations:** ^1^Shandong Academy of Agricultural Science, Jinan, China; ^2^Institute of Agro-Food Science and Technology, Shandong Academy of Agricultural Sciences, Jinan, China; ^3^The Department of Urology, The Shandong Provincial Third Hospital, Shandong University, Jinan, China; ^4^Institute of Poultry Science, Shandong Academy of Agricultural Sciences, Jinan, China

**Keywords:** *Flammulina velutipes*, dietary fiber, obesity, lipid metabolism, high-fat diet

## Abstract

**Introduction:**

Mounting evidence has shown that *Flammulina velutipes* mycorrhizae dietary fiber (Fv-DF) has the potential to significantly improve health outcomes by addressing lipid metabolic disorders. However, the mechanism underlying Fv-DF in regulating liver lipid metabolism of high-fat diet (HFD)-induced obese mice still merits to be systematically elaborated.

**Methods:**

Herein, we conducted a comprehensive study utilizing HFD-induced C57BL/6J mice as an obesity model to investigate the impact of Fv-DF on liver lipid accumulation.

**Results:**

The study, which included an evaluation of Fv-DF on a high-fat diet (HFD)-induced obese mice, revealed that Fv-DF supplementation can effectively decrease weight gain, improve serum lipid levels, and reduce fat deposition in adipose tissues. The estimation of Fv-DF on liver tissues demonstrated that Fv-DF supplementation significantly ameliorated lipid metabolism and hepatic injury in HFD-induced obese mice. Furthermore, Fv-DF improved lipid metabolism in obese mice by modifying the abundance and related pathways of TG, PC, PE, and other lipid metabolites. Mechanistically, Fv-DF supplementation significantly suppressed the expression of lipid synthesis-related genes while promoting lipid oxidation-related genes.

**Discussion:**

Collectively, the findings could inspire significant implications for Fv-DF in developing novel treatments for obesity-related metabolic disorders management.

## Introduction

The rapid advancement of the food production sector, particularly the increasing popularity of high-fat diets (HFD), has led to shifts in human lifestyle and eating patterns (1). However, numerous studies have demonstrated a strong negative correlation between HFD and human health, with the most immediate impact being the accumulation of organ fat and rising obesity rates (2). It has been documented that Chinese individuals’ fat consumption increased from 68.5 g/d to 79.3 g/d, resulting in a surge in obesity prevalence from 12.3 to 37.3% (3). According to the World Obesity Federation’s Atlas for 2023, an estimated global population of approximately 4 billion people (51%) will be affected by obesity or overweight within the next twelve years. Of concern is that obesity adversely affects most bodily functions and nearly all organ systems, leading to reduced lifespan and increased mortality rates (4). Existing pharmaceutical interventions and weight loss surgeries for preventing and managing obesity often entail various adverse effects (5). In contrast, lifestyle choices and adherence limit dietary regulation and physical activity. Therefore, there is a need to explore additional treatment targets and develop safer and more effective products for combating obesity.

The key feature of obesity is the disruption of lipid metabolism, with the liver playing a crucial role in regulating lipid intake, synthesis, and breakdown (6, 7). Fat accumulation from a high-fat diet can impair liver function and exacerbate glucose and lipid metabolism, leading to dyslipidemia and hepatic steatosis (8, 9). Therefore, analyzing changes in liver lipid metabolism is essential for managing and preventing obesity. Advanced detection technology has introduced high-resolution and high-sensitivity lipidomic analysis as an effective tool for comprehensively monitoring molecular-level lipid changes, replacing traditional biochemical detection methods (10). For instance, a study utilizing ultra-performance liquid chromatography–tandem mass spectrometry (UPLC-MS/MS) revealed that sea cucumber phospholipids increased nine types of phospholipids in mice with glomerulopathy caused by obesity induced by a high-fat diet, thereby ameliorating renal damage (11). A liver lipidomic analysis using UPLC-MS identified 11 potential lipid biomarkers in hyperlipidemic mice treated with *Gynostemma pentaphyllum* (12).

Using plant and fungal byproducts with functional food potential has attracted significant attention (13, 14). *Flammulina velutipes*, a widely cultivated edible mushroom in China, is highly regarded for its gastronomic appeal and substantial medical and healthcare benefits. Its mycorrhiza—a byproduct rich in biologically active compounds such as dietary fiber (DF) and polysaccharides—is particularly intriguing. Studies indicate that these components present in *F. velutipes* mycorrhiza exhibit antioxidant and immune-enhancing properties and hold promise for addressing lipid metabolic disorders (15–17). A prior investigation demonstrated that *F. velutipes* mycorrhiza DF (Fv-DF) can alleviate high-fat diet-induced obesity in mice through modulation of the intestinal microbial-mediated adenosine 5′-monophosphate (AMP)-activated protein kinase (AMPK) signaling pathway (18). Nevertheless, further exploration of the potential of Fv-DF to regulate liver lipid metabolism is warranted.

In this study, we utilized HFD-induced C57BL/6 J mice as an obesity model to investigate the impact of Fv-DF on lipid accumulation. We assessed serum lipid levels and adipose tissue indexes and performed biochemical and lipidomic analyses to explore hepatic function and lipid alterations. The results presented herein establish a basis for the potential application of Fv-DF in obesity management, suggesting promising prospects for its utilization in nutrition and obesity research.

## Materials and methods

### Dietary fiber preparation

The mycorrhiza utilized in the production of Fv-DF was sourced from the Institute of Agro-Food Science and Technology at Shandong Academy of Agricultural Science, China. The preparation process for Fv-DF followed a previously documented method (19). In brief, the mycorrhiza powder was dissolved in deionized water and subjected to hydrolysis with *α*-amylase and compound proteases separately at 55°C for 2 h. Following hydrolysis, the sample was precipitated by adding four times its volume of 95% ethanol and subsequently washed with acetone. The resulting precipitate underwent freeze-drying to yield the final Fv-DF product.

### Animal experiments

The study utilized 30 male C57BL/6 J mice, aged 4 weeks and weighing approximately 15 g, obtained from Huafukang Biotechnology Co. Ltd. (Beijing, China). The mice were housed in a controlled environment with a temperature of 22 ± 2°C, relative humidity of 55 ± 5%, and a 12 h light/dark cycle. After an acclimatization period of 7 days, the mice were randomly divided into three groups: chow diet (NF) group with a caloric density of 4.07 kcal/g and fat content of 5%, high-fat diet (HFD) group with a caloric density of 4.73 kcal/g and fat content of 45%, and HFD +10% FV-DF (Fv-DF) group (*n* = 10 per group). The HFD and Fv-DF groups were subjected to continuous HFD feeding for twelve weeks to induce obesity models. Lee’s obesity index was then calculated for each obese mouse model; an index exceeding 344.32 indicated the successful establishment of the obesity model (20). Subsequently, the HFD group transitioned to a chow diet, while the Fv-DF group switched to a chow diet supplemented with 10% Fv-DF for an additional 4 weeks, which 10% Fv-DF supplementation can effectively alleviate lipid metabolism disorder in our preliminary study (18). The NF group remained on the chow diet for sixteen weeks (18). Weekly mouse weight and food intake measurements were recorded throughout the experimental duration. On the last day of the experiment, fresh 24 h-faeces were collected and stored at −80°C. Then, humane euthanasia via cervical dislocation was performed on the mice; liver as well as groin and perirenal adipose tissues were collected post-sacrifice following ethical approval granted by the Ethics Committee of Shandong Academy of Agricultural Sciences (No. SAAS2021B01).

### Biochemical analysis

The levels of glycerides (TG), total cholesterol (TC), low-density lipoprotein cholesterol (LDL-C), and high-density lipoprotein cholesterol (HDL-C) in faeces, serum, and liver tissues were measured using a commercially available kit from Nanjing Jiancheng Bioengineering Institute, China, following the provided instructions.

### Histological analysis

The liver and perirenal fat tissue sections embedded in paraffin were stained with hematoxylin–eosin (H&E) (G1003, Servicebio, China) for microscopic examination. The inner diameter of adipose cells was measured using ImageJ software v. 1.54j (21). The groin fat tissues were preserved in optimal cutting temperature compound (OCT, Tissue-Tek 4,583, Sakura Finetek, United States), and the sections were stained with Oil-Red-O (O0625-25G, Merk Millipore, Germany) to observe lipid droplets.

### Lipidomic analysis

The lipid extraction and detection methods were described previously (22). In brief, 200 μL of pre-cooled water was added to liver samples from mice and homogenized at 4°C using an MP homogenizer. Subsequently, 800 μL of pre-cooled methyl tert-butyl ether and 240 μL of methanol were added, followed by vortexing for 30 s and sonication at 4°C for 20 min. The mixture was allowed to stand for 30 min at 10°C before being centrifuged at 14,000 g for 15 min. The upper organic layer was then dried in a vacuum centrifuge. The resulting dry sample was reconstituted in a solution of isopropanol/acetonitrile (v/v: 9:1) before lipidomic analysis.

For lipid extract analysis, the Q-EXACTIVE PLUS mass spectrometer (Thermo Fisher Scientific, United States) was employed with the UHPLC Nexera LC-30A (SHIMADZU, Japan). In brief, lipids were separated using a Waters Acquity Premier CSH C18 color spectrum column (1.7 μm × 2.1 mm × 100 mm). The chromatography conditions involved acetonitrile: water = 6:4, v/v as flow phase A and acetonitrile: isopropanol = 1:9, v/v as flow phase B, with a flow rate of 300 μL/min and a column temperature of 45°C. Gradient elution conditions included maintaining flow phase B at 30% from 0 to 2 min, increasing it from 30 to 100% from 2 to 25 min, and then returning it to 30% from 25 to 35 min. Electrospraying ionization (ESI) was used for mass spectrometry analysis under positive and negative ion modes. Full scanning spectra were collected in the 200–1800 m/z range for positive and negative ion modes. Peak extraction and lipid identification were conducted using LipidSearch software v. 4.1 (Thermo Fisher Scientific, United States).

Use Principal Component Analysis (PCA) and Orthogonal Partial Least-Squares-Discriminant Analysis (OPLS-DA) to visualize lipid changes in the HFD and Fv-DF groups. Differential lipid metabolites were identified based on the VIP scores obtained from the OPLS-DA model. Two-Tailed Student’s *t*-test was employed to calculate the metabolites’ Fold Change (FC) and *p* value. Metabolites meeting the criteria of VIP ≥ 1, |Log2 FC| ≥ 1, and *p* value ≤0.05 were considered significantly different metabolites. Differential metabolites were subjected to pathway enrichment analysis using MetaboAnalyst 6.0 (23).

### Western blot

Combine approximately 200 mg of liver tissue samples with 1 mL of RIPA Lysis Buffer containing 1 mM of proteinase inhibitor PSMF (Beyotime, China). Homogenize the mixture at −20°C for 30 min. Subsequently, the solution was centrifuged, and the supernatant was collected for subsequent western blot analysis. The protein concentration was determined using a BCA protein assay kit (Thermo Fisher Scientific, United States). Then, take an equivalent amount of protein and subject it to SDS-PAGE separation, transfer it onto a PVDF membrane, and block it using 5% nonfat milk at 4°C. Proceed by incubating the primary antibodies, including Scd1, Pparγ, Pparα, Pgc1, Cpt1b, and *β*-actin (Abcam, China) overnight at room temperature before incubating with the secondary antibody for 1 hour at room temperature. The last is to utilize the ECL system (Cell Signaling Technology, United States) to detect the protein signal. Greyscale analysis of Western blot bands was performed with ImageJ v 1.48 (NIH, United States).

### Quantitative real-time PCR

The total RNA was extracted from the liver tissue samples using the SteadyPure Universal RNA Extraction Kit according to the instructions provided (Accurate Biology, China). The quantitative real-time PCR (qRT-PCR) was performed according to what was described previously (17). In brief, 1 μg of total RNA was reverse transcribed to cDNA using the PrimeScrip RT Reagent Kit (Takara, Japan). The relative gene expression levels were determined by qRT-PCR using SYBR Premix EX Taq™ (Takara, Japan). *GAPDH* was the reference gene used to normalize the target gene expression levels. The relative gene expression levels were determined using a 2^−∆∆Ct^ method. All primer sequences used are listed in Supplementary Table S1.

### Statistical analysis

The data were presented as the mean ± standard deviation (SD). The data normality was performed with the Kolmogorov–Smirnov test, where *p* > 0.05 fit a normal distribution. For data with a normal distribution, one-way ANOVA with the Tukey *post hoc* test was used to compare the differences between groups, and for data that failed the normality tests, the Mann–Whitney U test was used in GraphPad Prism 9.0 (GraphPad Software, Inc., United States). Statistical significance was considered at a *p* value ≤0.05.

## Results

### Fv-DF decreased fat accumulation in HFD-induced obese mice

**Figure 1:**
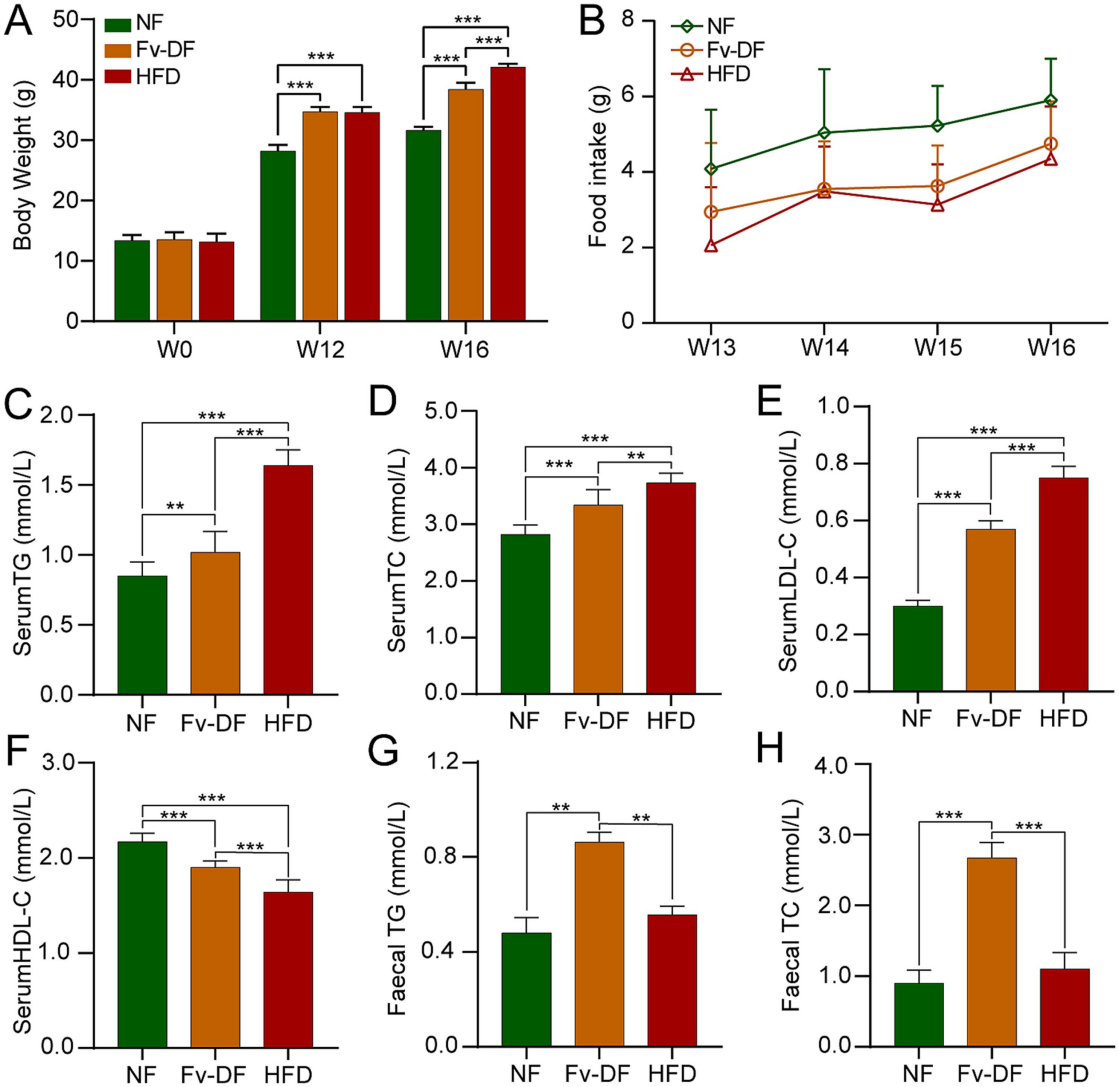
Fv-DF reduced body weight gain and improved lipid metabolism in HFD-induced obese mice. **(A)** Body weight changes of mice during experiment in three groups. **(B)** Food intake in mice with Fv-DF or HFD supplementation (Repeated measures ANOVA, main effect of diets, *F*_2,9_ = 4.520, *p* = 0.061). **(C–F)** Serum TG **(C)**, TC **(D)**, LDL-C **(E)**, and HDL-C **(F)** levels were analyzed by ELISA. **(G,H)** Fecal TG **(G)** and TC **(H)** levels. Data are expressed as mean ± SD. One-way ANOVA followed by Tukey *post hoc* test for pairwise comparisons. ** *p* < 0.01, *** *p* < 0.001.

To confirm the beneficial impact of Fv-DF on HFD-induced obesity, we employed C57BL/6 J mice as an obesity model and subjected them to HFD. The mice were continuously fed HFD for 12 weeks, followed by a switch to a chow diet containing 10% Fv-DF for 4 consecutive weeks. At the beginning of the experiment, there were no significant differences in weight gain among the groups. After 12 weeks of HFD feeding, the HFD and Fv-DF groups exhibited an average weight exceeding 35 g, significantly higher than that of the NF group mice. Lee’s obesity index exceeded 344.32, indicating successful modeling of nutritional obesity in mice (Figure 1A). Following 4 weeks of Fv-DF treatment, it was revealed that compared to the HFD group, the weight gain of the Fv-DF group had significantly decreased (Figure 1A). Furthermore, the average food intake of the NF group mice was slightly higher than that of those fed HFD, suggesting its effect on mouse appetite; however, there was no significant difference between food intake in both HFD and Fv-DF groups (Figure 1B). These results suggest that Fv-DF can effectively ameliorate HFD-induced obesity without affecting appetite.

Lipid metabolic disorders are a significant consequence of obesity resulting from a high-fat diet. Compared to the NF group, the serum TG, TC, and LDL-C levels were notably elevated in the HFD group (Figures 1C–E), while HDL-C level was significantly reduced (Figure 1F). Fv-DF exhibited a significant reduction in TG, TC, and LDL-C levels compared to the HFD group and increased HDL-C levels. Furthermore, the fecal TG and TC levels were significantly increased in the Fv-DF group compared to the HFD group (Figures 1G,H).

### Fv-DF mitigated lipid metabolic disorders in HFD-induced obese mice

**Figure 2:**
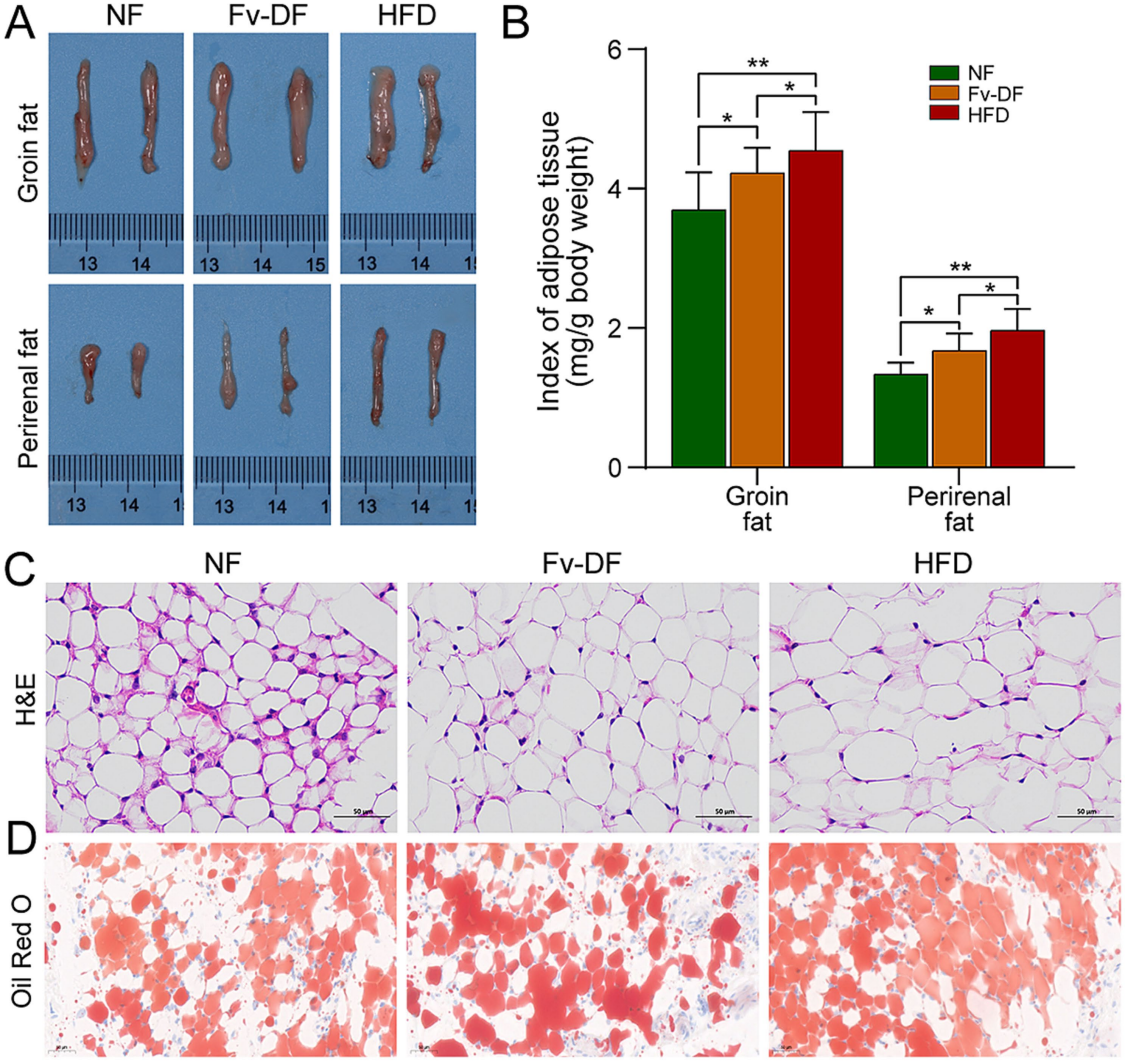
Fv-DF improved fat deposition in HFD-induced obese mice. **(A)** Groin fat and perirenal fat tissues. **(B)** Index of adipose tissue for groin fat and perirenal fat. **(C)** Perirenal fat histology on H&E straining sections (scale bar, 50 μm). **(D)** Groin fat histology on Oil-Red-O straining sections (scale bar, 50 μm). Data are expressed as mean ± SD. One-way ANOVA followed by Tukey post hoc test for pairwise comparisons. * *p* < 0.05, ** *p* < 0.01, *** *p* < 0.001.

Analyzing alterations in primary fat tissues showed that the accumulation of groin fat and perirenal fat was significantly higher in the HFD group than in the NF group (Figure 2A), leading to a larger adipose tissue index (Figure 2B). Pathological analysis using H&E staining revealed that compared to the NF group, perirenal fat cells were generally larger and contained larger lipid droplets within them for the HFD group (Figure 2C). Conversely, compared with the HFD group, it was noted that Fv-DF resulted in uneven sizes of perirenal fat cells with significantly smaller lipid droplet diameters. Consistent with this observation, Oil-Red-O staining results indicated that compared to the HFD group, the Fv-DF significantly reduced the number of lipid droplets in the groin fat tissue (Figure 2D). These results suggested that Fv-DF can promote fat metabolism, inhibit fat accumulation, and improve the cell size of adipose tissue, thereby achieving a certain anti-obesity effect.

### Fv-DF ameliorates hepatic injury in obese mice and enhances lipid metabolism

**Figure 3:**
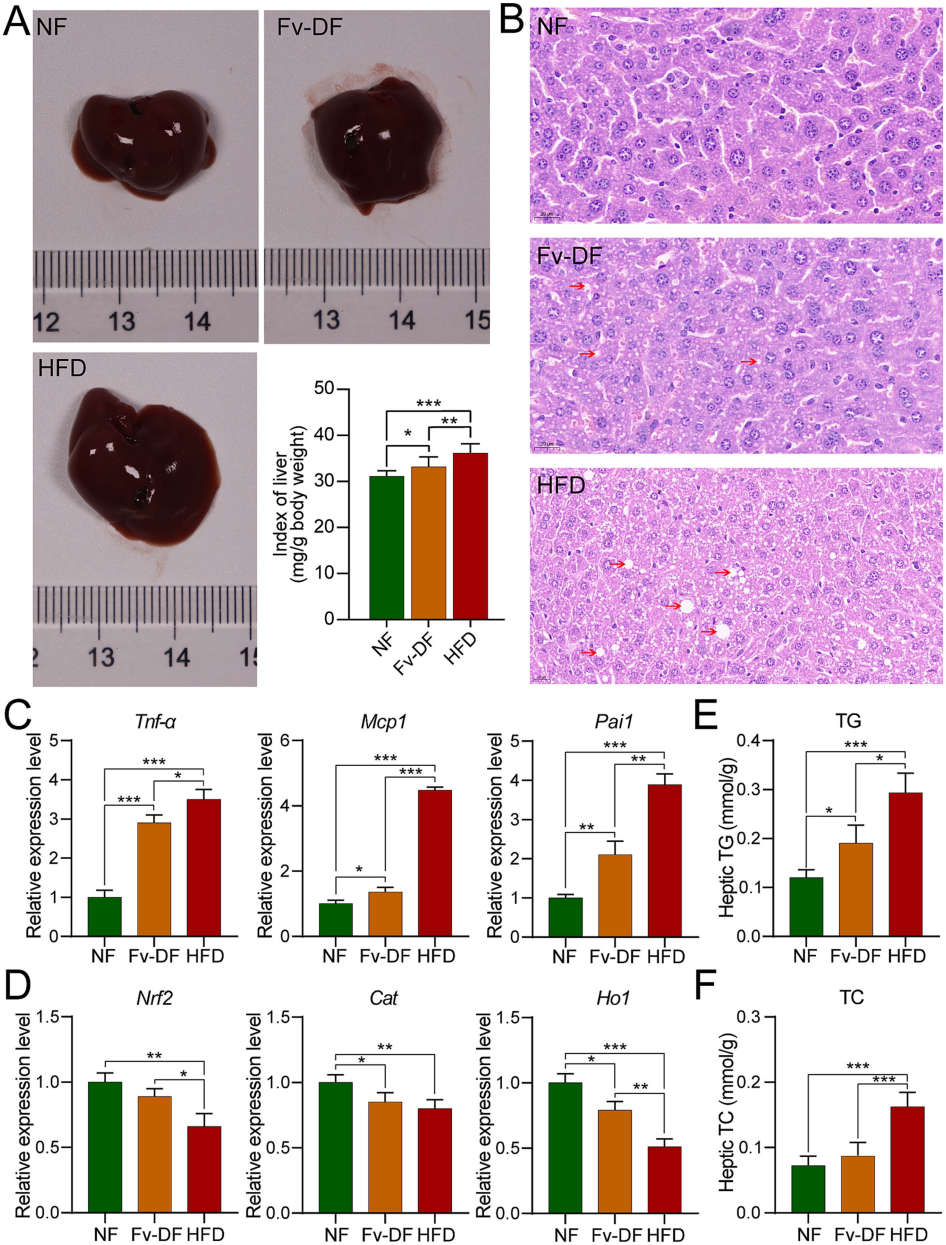
Fv-DF improved liver damage and lipid metabolism in HFD-induced obese mice. **(A)** Liver tissues and index. **(B)** H&E staining of liver tissues (scale bar, 20 μm). **(C,D)** Relative mRNA expression level of inflammatory cytokines **(C)** and antioxidant genes **(D)** in liver tissues. **(E,F)** Levels of liver TG **(E)** and TC **(F)**. Data are expressed as mean ± SD. One-way ANOVA followed by Tukey post hoc test for pairwise comparisons. * *p* < 0.05, ** *p* < 0.01, *** *p* < 0.001.

The liver was subjected to morphological and pathological analyses as the primary organ responsible for regulating lipid metabolism. As shown in Figure 3A, there was no significant difference in liver appearance among different groups. However, the liver index of the HFD group was significantly higher than that of the NF group, while the liver index of the Fv-DF group significantly recovered and decreased compared with the HFD group. H&E staining results showed that compared with the normal liver cells in the NF group, those in the HFD group exhibited white spot-like fat vacuoles around them, indicative of hepatic steatosis. Compared with the HFD group, the fat vacuole phenomenon in the Fv-DF group was reduced (Figure 3B), suggesting that Fv-DF can delay high-fat diet-induced hepatic steatosis and provide partial protection for the liver.

Obesity is typically characterized by low-grade chronic inflammation in adipose tissue, common in visceral fat. Compared with the NF group, the mRNA expression levels of pro-inflammatory cytokines such as *Tnf-α*, *Mcp1*, and *Pai1* secreted by hepatic adipocytes were significantly elevated in the HFD group. Notably, these expression levels were restored towards normalcy or reduced upon supplementation of Fv-DF compared to those observed in the HFD group (Figure 3C).

Furthermore, the accumulation of adipose tissue can cause excessive oxidative damage to the body. As shown in Figure 3D, compared with the NF group, the mRNA expression levels of antioxidant stress-related genes *Nrf2*, *Cat*, and *Ho1* were significantly decreased in the HFD group. Compared with the DF group, the Fv-DF group partially restored the expression of these genes associated with oxidative stress response. These findings indicate that Fv-DF alleviates the inflammatory response of adipose tissue caused by the HFD diet and improves the antioxidant capacity of obese mice.

To further verify whether Fv-DF alleviates liver lipid metabolism, we measured the total contents of TG and TC in the liver (Figures 3E,F). Compared with the NF group, the levels of TG and TC were significantly elevated in the HFD group. Conversely, compared with the HFD group, the Fv-DF group significantly reduced the contents of TG and TC, hence alleviating liver lipid metabolism. These results collectively demonstrate that Fv-DF ameliorates hepatic injury in HFD-induced obese mice and enhances lipid metabolism.

### Identification of biomarkers related to hepatic lipid metabolism

**Figure 4:**
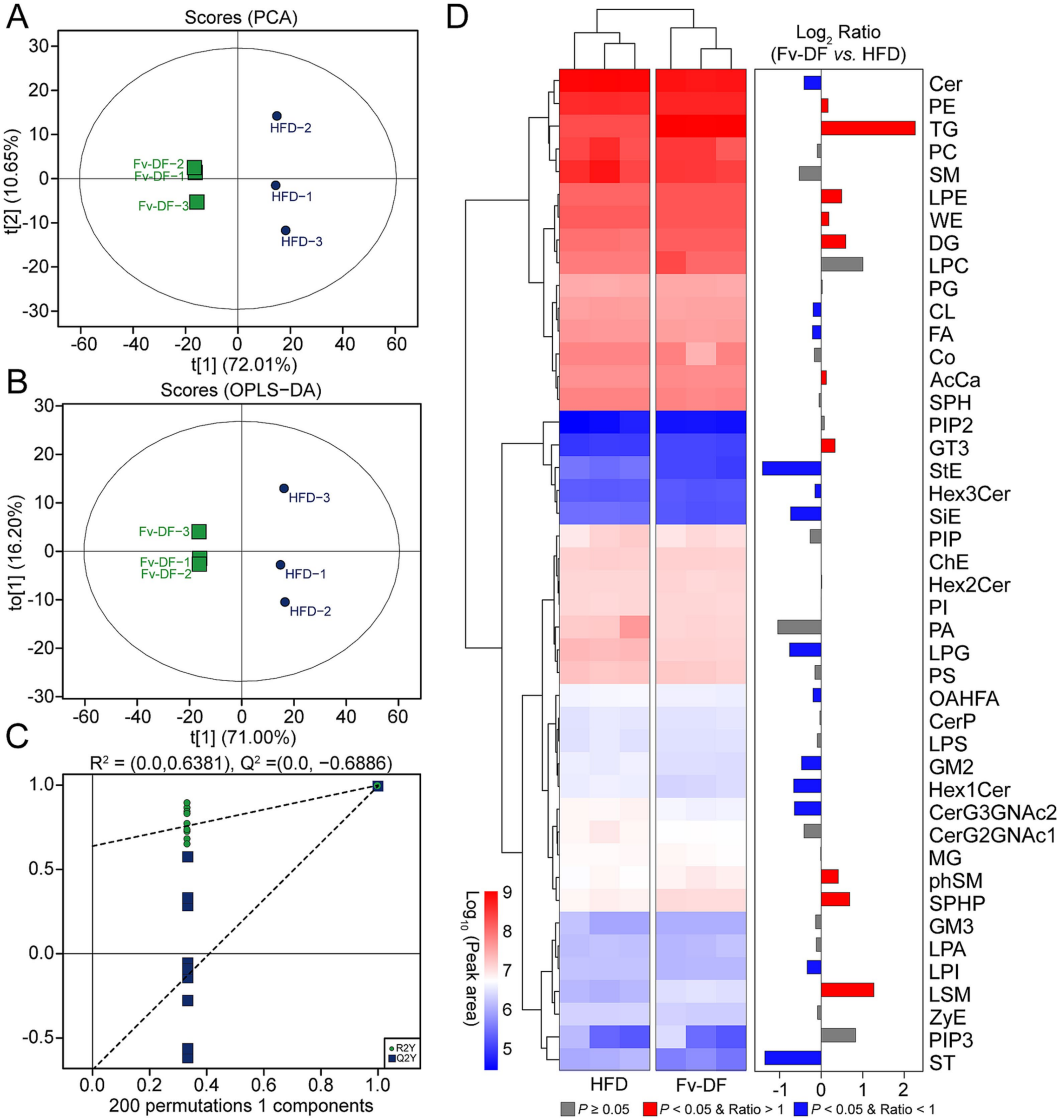
Liver lipidomic profiles of HFD-induced obese mice treated with HFD or Fv-DF. **(A,B)** PCA plot **(A)** and OPLS-DA plot **(B)** of the mice in HFD and Fv-DF groups. **(C)** Permutation test of OPLS-DA models. **(D)** Differential lipid classes between the Fv-DF group and the HFD group. The heatmap shows the relative expression level of the lipids, and the bar chart shows the fold changes between the two groups.

To investigate changes in hepatic lipid metabolism and the potential relationship between key lipid biomarkers and the biological activity of Fv-DF, we employed lipidomic to analyze distinct lipid compositions in the HFD and Fv-DF groups. A total of 44 classes comprising 2,158 liver lipid metabolites were identified, including 500 triglycerides (TGs), 274 ceramides (Cers), 247 phosphatidylethanolamines (PEs), 221 phosphatidylcholines (PCs), 181 diglycerides (DGs), 74 cardiolipins (CLs), 69 phosphatidylglycerols (PGs), 68 sphingomyelins (SMs), 61 phosphatidylserines (PSs), and 59 wax esters (WEs). Principal component analysis revealed clear group separation between the HFD group and the Fv-DF group (Figure 4A). Similarly, OPLS-DA analysis also demonstrated consistent results (Figure 4B). The statistical model analysis indicated good adaptability and predictability for distinguishing between the HFD and Fv-DF groups (Figure 4C). This suggests that the OPLS-DA model can effectively differentiate between these two groups and serve as a predictive tool for evaluating alterations in lipidomic profiles. Furthermore, comparative analysis revealed significant increases in TG, lysophosphatidylethanolamine (LPE), WE, and DG, while Cer, CL, fatty acid (FA), and lysophosphatidylglycerol (LPG) were significantly decreased within the Fv-DF group compared to the HFD group (Figure 4D).

**Figure 5:**
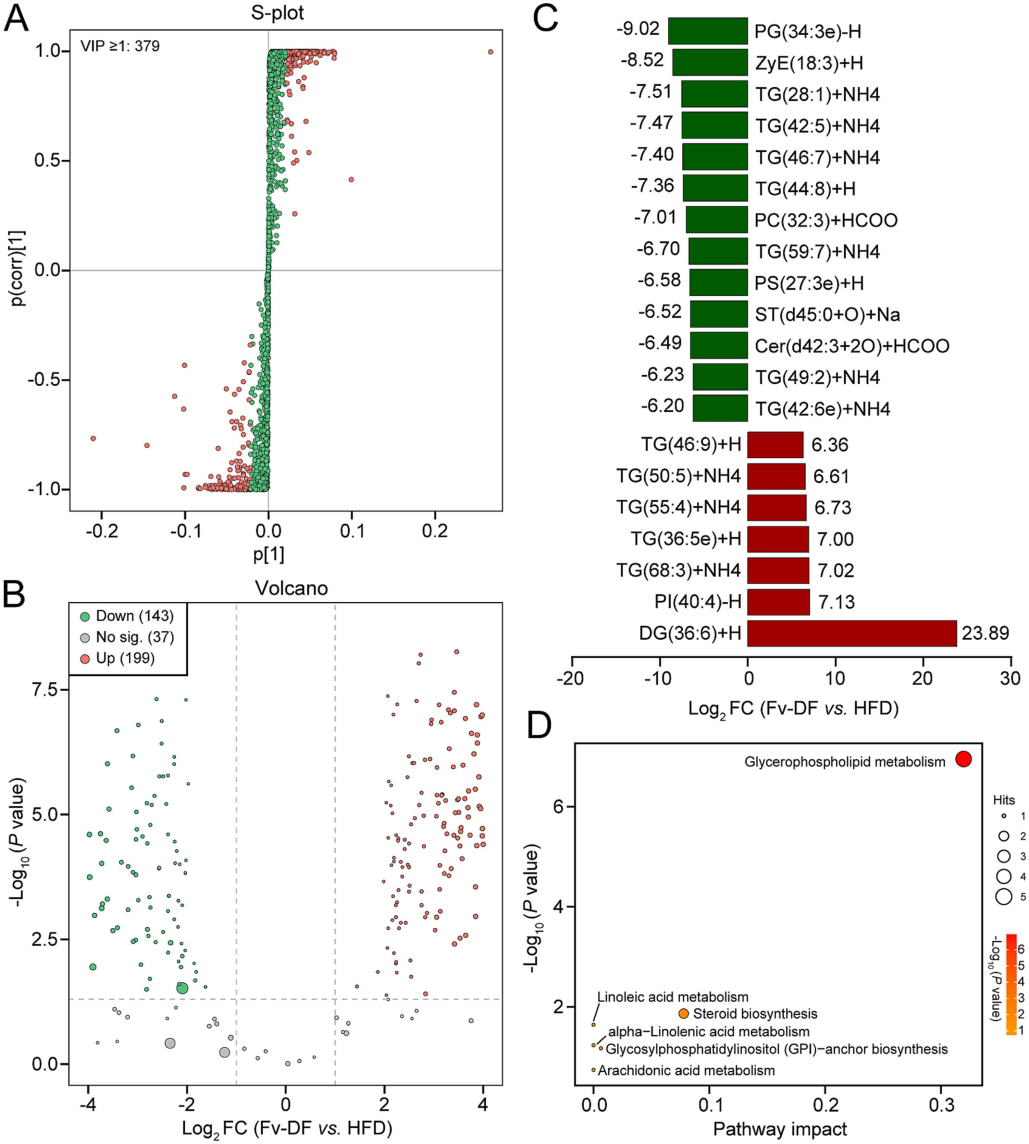
Screening and enrichment analysis of differential lipid metabolites. **(A)** S-plot showing the differential lipid metabolites based on VIP ≥ 1 between the Fv-DF and DF groups. **(B)** Volcano plot showing differential lipid metabolites based on *p* value ≤0.05, |Log2FC| ≥ 1, and VIP ≥ 1. **(C)** Bar chart showing the top 20 differential lipid metabolites with the largest fold changes. **(D)** Dot plot of KEGG enrichment analysis for differential lipid metabolites.

The VIP score calculated from the OPLS-DA model is a crucial indicator for assessing differential metabolites. Compared to the HFD group, 379 differential metabolites with VIP ≥1 were identified in the Fv-DF group (Figure 5A). Based on the |Log2 FC| ≥ 1 and *p* value ≤0.05, a total of 342 differential lipid metabolites were further identified in the Fv-DF group, including199 up-regulated and 143 down-regulated (Figure 5B; Supplementary Table S2). The classification of these differential lipid metabolites revealed 179 TGs (36 down-regulated), 57 DGs (23 down-regulated), 20 Ceramides (15 down-regulated), 17 Phosphatidylcholines (17 down-regulated), 14 Phosphatidylserines (12down-regulated), and 11 Phosphatidylethanolamines (9 down-regulated). Of the topmost significant 20 differential metabolites, 12 lipid metabolites were down-regulated in the Fv-DF group, including 7 TGs, PG, ZyE, PC, PS, and ST (Figure 5C). Furthermore, KEGG pathway enrichment analysis showed that the differential lipid metabolites were primarily associated with glycerophospholipid metabolism, linoleic acid metabolism, and steroid biosynthesis (Figure 5D). These findings suggest that Fv-DF improves lipid metabolism in HFD-induced obese mice by modifying the abundance and related pathways of TG, PG, PE, and other lipid metabolites.

### The potential mechanism of Fv-DF improving lipid metabolism in HFD-induced obese mice

**Figure 6:**
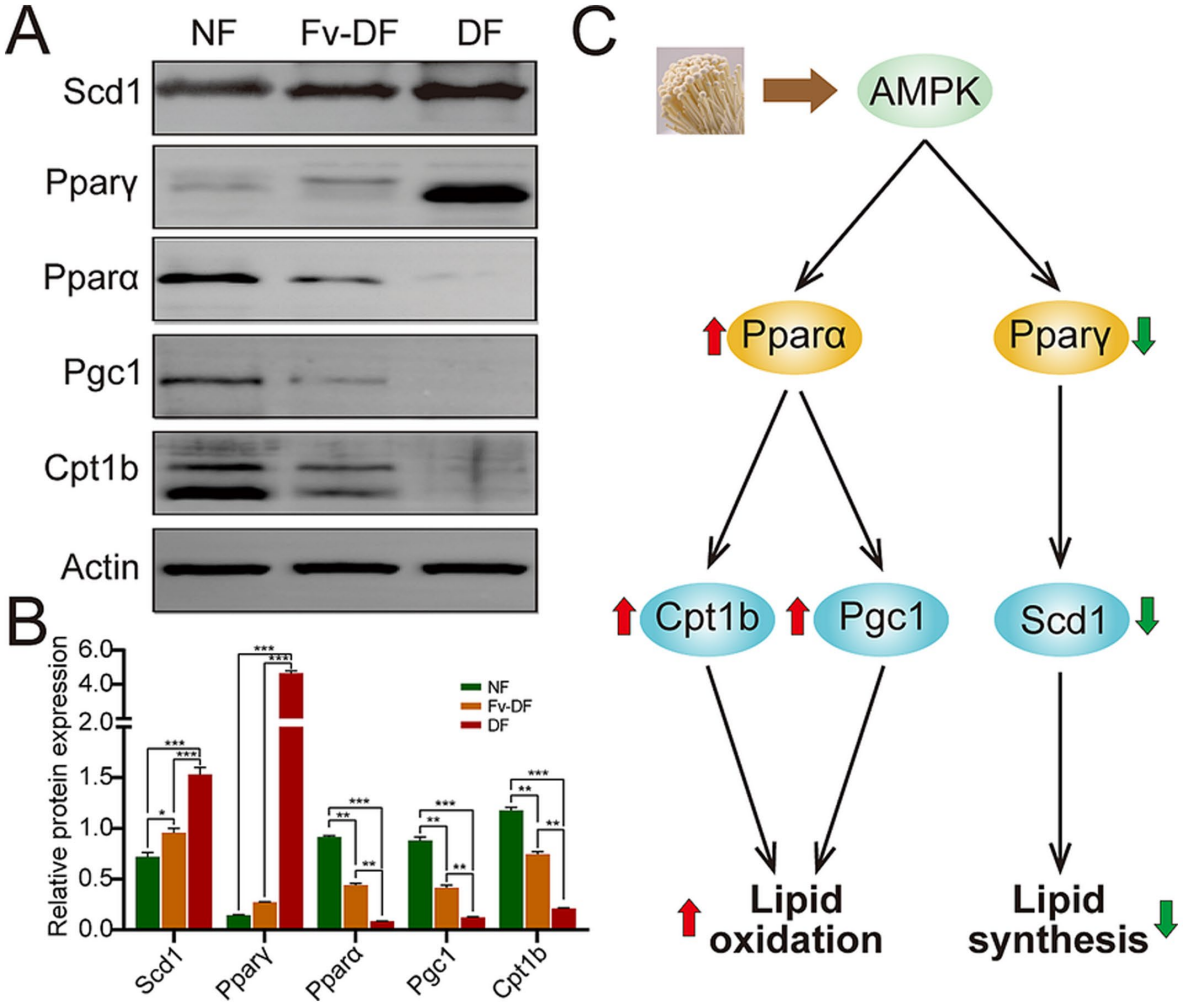
The potential mechanism of Fv-DF in treating HFD-induced obese mice. **(A,B)** Western blot analysis of lipid oxidation and synthesis-related proteins **(A)** and band intensities were estimated against the control Actin **(B)**. **(C)** The potential mechanism of Fv-DF in the treatment of HFD-induced obese mice. Fv-DF supplementation down-regulated the expression level of the lipid synthesis-related gene Scd1 and up-regulated the lipid oxidation-related gene Cpt1b and Pgc1 to improve lipid metabolism in HFD-induced obese mice. Data are expressed as mean ± SD. One-way ANOVA followed by Tukey post hoc test for pairwise comparisons. * *p* < 0.05, ** *p* < 0.01, *** *p* < 0.001.

Lipid oxidation and lipid synthesis are two crucial processes that regulate lipid metabolism; we explored several pivotal proteins involved in the two processes among NF, HFD, and Fv-DF groups (Figures 6A,B). Compared with the NF group, HFD resulted in elevated expression of Pparγ and Scd1 proteins in the liver of obese mice in the HFD group, while it reduced the expression of Pparα, Pgc1, and Cpt1b proteins. In contrast to the HFD group, Fv-DV supplementation suppressed the expression of Pparγ and Scd1 while enhancing the expression of Pparα, Pgc1, and Cpt1b. These findings collectively suggest that Fv-DF likely mitigates lipid accumulation in HFD-induced obese mice by promoting lipid oxidation and inhibiting lipid synthesis (Figure 6C).

## Discussion

The present study delved into the potential of Fv-DF to be an effective intervention for mitigating lipid accumulation in HFD-induced obese mice. Our findings, based on serum lipid levels, adipose tissue indexes, and biochemical analyses, suggest that Fv-DF has the potential to be an effective intervention for mitigating lipid metabolic disorders and hepatic injury in obese mice while enhancing lipid metabolism. Furthermore, lipidomic analysis on liver tissues revealed that Fv-DF improves lipid metabolism in obese mice by modifying the abundance and related pathways of TG, PC, PE, and other lipid metabolites. We also uncovered the potential action mechanism of Fv-DF in improving lipid metabolism in HFD-induced obese mice, which is likely by promoting lipid oxidation and inhibiting lipid synthesis. These findings could inspire significant implications for the development of novel treatments for obesity-related metabolic disorders.

In this study, we first assessed the impact of Fv-DF on weight gain in HFD-induced obese mice. The results showed that Fv-DF significantly decreased the weight gain in HFD-induced obese mice without affecting appetite compared to the HFD group. These findings, which align with previous studies (8, 24), suggest that the impact of Fv-DF on weight gain in obese mice is likely associated with lipid breakdown and metabolism. The levels of TC and TG in fecal samples were determined to confirm the conclusion, and the results revealed that both levels were significantly increased in the Fv-DF group compared to the HFD group, indicating a promoting role of Fv-DF in excess lipid excretion from the intestinal tract of the obese mice. The determination of TC and TG levels in liver tissue also showed that both were lower in the Fv-DF group than in the HFD group, suggesting a crucial role of Fv-DF in alleviating the occurrence of lipotoxicity (25, 26). These findings collectively suggest that Fv-DF supplementation can effectively mitigate lipid metabolic disorders in HFD-induced obese mice, offering a promising avenue for future research and potential therapeutic interventions. However, more accurate methods such as magnetic resonance imaging (MRI) and dual-emission X-ray absorptiometry (DEXA) are needed to examine changes in total body fat mass in mice to assess the actual effects of Fv-DF on fat metabolism.

Our study, which investigated hepatic lipid alteration in HFD-induced obese mice with Fv-DF supplementation, is significant due to the liver’s pivotal role in lipid metabolism. We identified 2,158 liver lipid metabolites and 379 differential metabolites between the Fv-DF and HFD groups. PE and PC, known lipid species in the normal obese liver (27), are crucial in promoting adipogenesis and increasing lipid droplet and TG levels (28). This study identified 11 differential PEs and 17 differential PCs in the Fv-DF group compared to the HFD group. Most PE and PC metabolites decreased in the Fv-DF group, aligning with liver morphological and pathological analyses. This suggests a critical role of Fv-DF supplementation in reducing liver adipogenesis and lipid droplet accumulation in HFD-induced obese mice, indicating its potential therapeutic implications. In this study, the trends in TG and DG species varied, with most of the differential TGs and DGs up-regulated in the Fv-DF group. The results aligned with several previous reports (29, 30), but the occurrence of these contrast observations may vary from different obesity models used (31). Moreover, the pathway enrichment analysis revealed that these differential lipid species were primarily related to glycerophospholipid metabolism, linoleic acid metabolism, and steroid biosynthesis. These findings open up intriguing possibilities for the potential role of Fv-DF supplementation in managing hepatic lipid alteration in obesity.

Lipid oxidation and synthesis are two crucial processes that regulate lipid metabolism. This study explored several pivotal proteins involved in the process among NF, HFD, and Fv-DF groups to investigate the potential mechanism underlying Fv-DF supplementation in improving lipid metabolism in HFD-induced obese mice. More evidence has shown that the elevation of Pparγ and Scd1 proteins were correlated with the increase of hepatic lipid synthesis (32–34); our study showed that the Fv-DF supplementation significantly suppressed the protein expression levels in HFD-induced obese mice. Furthermore, the investigation of Pparα, Pgc1, and Cpt1b, which were involved in lipid oxidation (35, 36), showed that the expressions of these proteins were remarkably elevated in liver tissue in HFD-induced obese mice upon Fv-DF supplementation. These findings collectively indicated that the anti-obesity of Fv-DF supplementation in HFD-induced obese mice promotes lipid oxidation and inhibits lipid synthesis.

## Conclusion

In summary, this study systematically explored the anti-obesity of Fv-DF supplementation in HFD-induced obese mice. The findings showed that Fv-DF supplementation can effectively improve lipid metabolic disorders in HFD-induced obese mice, largely by promoting lipid oxidation and inhibiting lipid synthesis. The results presented herein establish a basis for the potential application of Fv-DF in obesity management, suggesting promising prospects for its utilization in nutrition and obesity research.

## Data Availability

The original contributions presented in the study are included in the article/Supplementary material, further inquiries can be directed to the corresponding author.
